# Impact factors of Arctic research stations on the mental health of team members

**DOI:** 10.3389/fpsyg.2025.1567378

**Published:** 2025-05-07

**Authors:** Huibao Li, Guangtian Zou

**Affiliations:** School of Architecture and Design, Harbin Institute of Technology, Key Laboratory of Cold Region Urban and Rural Human Settlement Environment Science and Technology, Ministry of Industry and Information Technology, Harbin, China

**Keywords:** Arctic research station, mental health, restorative environment theory, design factors, principal component analysis, factor weight

## Abstract

**Introduction:**

The extreme, closed, and isolated environments of Arctic research stations have resulted in substantial challenges in the daily life and work of polar science expedition team members, often leading to various mental health problems. The mental health of the Arctic team members is related to the restorative quality of the station environment, which is primarily influenced by design factors. However, previous studies have mainly discussed these factors separately using a single approach, rarely providing comprehensive understanding into team members' perceptions of the station environment in an integrated manner.

**Methods:**

This study uses a combination of qualitative and quantitative methods to determine the design factors and dimensions that affect the restorative potential of Arctic research stations, filling the gap in the design weight of restorative potential factors. First, environmental factors related to the mental health of team members in Arctic research stations were gradually screened through a literature review and semi-structured interviews. Then, questionnaire was used to collect the perspectives of 63 Chinese scientific research team members. Correlation analysis, principal component analysis, and statistical weight calculation were applied to the responses to investigate the restorative quality of design characteristics within a framework.

**Results:**

The findings showed that 24 design factors were associated with a restorative scientific research station environment. Among them, privacy of space was the most significant factor that could influence psychological recovery. Five primary components were identified: indoor conditions, configuration of space, physical environment, spatial perception, and space safety. Additionally, nine secondary components were identified: appearance design, spatial scale, interior facilities, space function, outdoor environmental influences, room adjustments, personal contact, interaction, and space safety. The weight calculation shows that indoor conditions and space configuration are the most significant dimensions that influence the restorative quality.

**Discussion:**

This study provides a targeted analysis of the environmental factors and key dimensions of Arctic research stations, offering a theoretical basis and practical suggestions for architectural design of Arctic research stations that are more suitable for team members.

## 1 Introduction

The harsh natural environment of polar regions (Bishop et al., 2010; Suedfeld, 1991; Wolak and Johnson, 2021), and the closed, isolated social environment of the research stations (Carrere and Evans, 1994; Michel et al., 2015; Nicolas et al., 2016; Palinkas et al., 1998) have brought great challenges for mental health to the research team members who live, work, and study. Psychologists first investigated the symptoms of them (Kokun and Bakhmutova, 2020; Leon et al., 2011; Universidade Federal Do Rio, Grande Brasil, 2013), they have found that the most common symptom among polar team members was a higher incidence of negative psychological outcomes (Hawkes and Norris, 2017), including depression, sleep disturbance, irritability, and a decline in motivation (Alfano et al., 2021; Chengli et al., 2003; Cochrane and Freeman, 1989; Johnsen et al., 2012; Palinkas and Houseal, 2000; Van Ombergen et al., 2021). Although with recent improvements in research station, team members have begun to experience more positive emotions (Bishop et al., 2010; Steel and Suedfeld, 2016), however, existing studies still neglect to explore the restorative resources generated by team members in coping with stress. Therefore, it is urgent to carry out adaptive restorative design for research station resources to effectively improve the mental health of team members. Therefore, polar research stations should be transformed into resources to help the team members better cope with their extremely closed conditions and to develop rational environmental health intervention strategies to promote mental restoration (Kearns and Moon, 2002).

Existing restoration theories include Stress Reduction Theory (SRT) (Ulrich et al., 1991) and Attention Restoration Theory (ART) (Kaplan, 1995). Stress Reduction Theory (SRT) points that stress is omnipresent in work and life, and it claims that exposure to nature supports psychophysiological stress recovery. In ART, directed attention is a core element of ART, Kaplan suggests that restorative environments emphasize the connection between people and their surroundings (Dean et al., 2018). The two theories point out appropriate environmental design improves psychological wellbeing by reversing stress responses, fostering positive emotions after a stressful experience, and reducing mental resource consumption (Hartig and Staats, 2003). Notably, four factors have been identified as characterizing restorative environments: being away, fascination, extent, and compatibility (Kaplan, 1995). Being away does not necessarily refer to a pleasant place, but instead to a respite from routine tasks that are tiresome and monotonous. Fascination refers to effortless attention directed at interesting content; it is the intensity of fascination that allows an individual to remain captivated for as long as possible, delaying a return to stress-inducing thoughts. Extent refers to the perception of the environment, encompassing not only to interesting places or things, but also to processes such as thinking, doing, and wandering. Compatibility is the balance between one's inclinations, the activities they want to pursue, and the environment. In terms of the polar research station, design factors are regarded as mediators of its architecture's influence on team members restorative outcomes, this situates design factors as significant features that affect team members restoration.

Unlike traditional buildings, the design of polar stations need to consider how to deal with unique challenges within extreme climatic conditions and the limited space and resource constraints, this situates design characteristics as significant features that affect restoration (Davis, 2017; Temp et al., 2017; Yan and England, 2001). Environmental characteristics such as extreme temperature, snow cover and strong photoperiod pose severe challenges to the thermal comfort and lighting design of buildings (Abazari et al., 2022). Therefore, the researchers first paid attention to the relationship between building thermal comfort, light regulation and human comfort. Adjustable artificial lighting systems can simulate changes in natural light and support circadian rhythm regulation (Jakubiec, 2023). Subsequently, researchers found that incorporating natural elements into the built environment can have a positive impact on the biological health of occupants (Joye, 2007). This also applies to Arctic dwellers (Parsaee et al., 2021), particularly in terms of reducing stress and anxiety, improving mental performance, and regulating the biological clock (Sarris, 2017). As extreme environments limit people's outdoor activities (Hassi et al., 2005), researchers pay more attention to the interaction between natural elements and indoor environment. For example, natural elements such as light and wood provide a sense of security and satisfaction (Bannova and Nyström, 2015; Panagopoulos et al., 2021). Additionally, Odeh and Guy (2017) demonstrated that engaging in gardening activities during missions can activate brain regions, thereby promoting mental health. Subsequently, researchers gradually shifted their attention from indoor natural elements to the interior design features. Polar research stations need to meet the needs of different individuals and groups in limited space and resources to maximize recovery benefits. Therefore, for the group, the spatial structure and multi-functional design of space are particularly important. Larger spaces generally promote a sense of freedom and relaxation, while crowded and confined spaces can lead to feelings of oppression and anxiety, potentially harming the mental health and wellbeing of team members (Connors et al., 1985). The multi-functional design can enhance the diversity and adaptability of the space. In a visually monotonous polar environment, the aesthetics and comfort of the polar research station significantly impact team members' health and wellbeing (Harrison et al., 1989; Wolak and Johnson, 2021). For example, color stimuli can trigger emotional changes in team members (Palinkas et al., 1998; Parsaee et al., 2019). For individuals, meeting smaller environmental resources or personal needs can lead to restoration experiences. Personalized living spaces, and private design can significantly enhance the mental health. Previous studies on the potential restorative quality of polar research stations mainly focuses on various design characteristics. Which are briefly listed in Table 1.

**Table 1:** Design characteristics that have potential restorative quality in polar research stations.

**Design factors**	**Effect on mental health**	**Design factors**	**Effect on mental health**
**Natural light illumination**	The light of the station environment impacts members' health by affecting sleep quality (Espinoza-Sanhueza et al., 2024; Jakubiec, 2023).	**Color**	The polar colors are monotonous, and the stimulation of colors can trigger changes in the emotions of the members (Palinkas et al., 1998; Parsaee et al., 2019).
**Size of room** **Room height**	The size and height of the room affect mental health through perception. Cramped and confined environments can have a serious impact on the health and wellbeing of team members (Connors et al., 1985; Peralta Martin-Palomino, 2022).	**Window size**	The natural element is the main restorative factor for mental health. The size of the window is related to the natural scenery outside and the amount of natural light that enters, which can affect mood (Korpela et al., 2017).
**Material**	The gentle nature of wood as a factor in the natural environment gives team members special security and satisfaction (Nyrud and Bringslimark, 2010).	**Furniture design**	Good furniture design can reduce the waste of indoor space and crowding. Humanized furniture affects the physical experience of the team members, thus affecting their mental health (Simon and Toups, 2014).
**Temperature humidity**	The physiological changes in the body created by increasing the temperature (Parkinson et al., 2020) and humidifying the air affect the mental health of the team members (Yan and England, 2001).	**Sound**	Polar winds and indoor mechanical sounds affect the mental health of team members by affecting their sleep quality (Bannova and Nyström, 2015).
**Privacy of the space** **Personalized setting of space**	Proper control of privacy can improve team members' satisfaction and cultivate an emotional connection with the environment (Jaksic et al., 2020). Personal space settings can enhance the members' sense of space domain and control, thus affecting their mental health (Davis, 2017).	**Space to exercise**	Exercise itself is good for physical and mental health. Increasing sports and recreational activities helps relieve pressure and generate positive emotions (Abeln et al., 2015)
**Novelty, soft decorations**	Beautiful and soft design can cultivate a positive mood in the team members and promote their wellbeing (Harrison et al., 1989).	**Smell** **Air** **Ventilation**	Ventilation and cleanliness ensure that experimental items will not produce flammable or explosive situations and enhance comfort by providing a safe, sanitary environment (Davis, 2017).
**Gardening activities**	Gardening activities during missions can activate brain regions, thereby promoting mental health (Odeh and Guy, 2017).	**Functional zoning of the space**	The multi-functional design can enhance the diversity and adaptability of the space (Bannova and Nyström, 2015).

The polar symptoms experienced by team members underscore the significance of the restorative perception of the indoor environment within research stations. Consequently, restorative theory is central to interpreting the impact of Arctic conditions on team members' health. However, the relationship between restorative quality and design factors in Arctic research stations has not been thoroughly studied. Current research has evaluated single design factors from various perspectives and using different indicators, with qualitative research methods have been adopted to explore factors related to team members' wellbeing, encompassing a wide range of aspects. However, the architectural factors investigated is limited, and the methods used relatively uniform. Therefore, this study aims to apply restorative environment theory, employing a mixed-methods approach that combines qualitative and quantitative research to identify key factors influencing the restorative perception of spaces within Arctic research station, and to explore the intrinsic structure of these elements, thereby filling the gap in the design weight of restorative factors. This will enable them to perform their tasks more efficiently and provide design insights for the architecture planning of Arctic research stations. More specifically, this study aims to determine the following: (1) What are the architectural design factors that affect the team members' perceived restorative quality of the indoor environment in the Arctic research station? (2) What is the underlying structure of key restorative design factors in Arctic research stations, and how can they be prioritized for optimal mental health support?

## 2 Research methods

### 2.1 Research design

**Figure 1:**
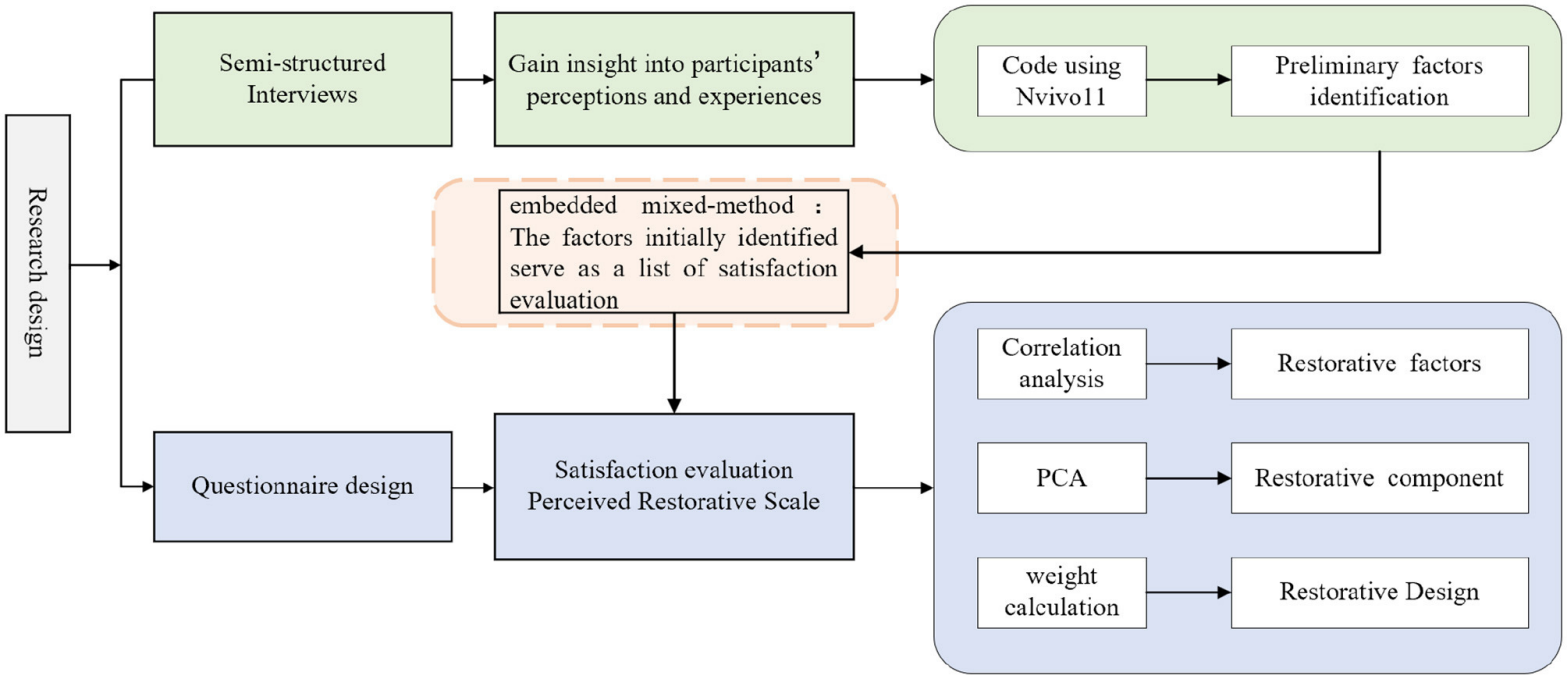
Arctic Research Stations restorative environment research framework.

This research used an embedded mixed-methods design. A mixed research approach enables the collection, analysis, and mixing of quantitative and qualitative data in a study or a series of studies to more completely understand the research problem (Johnson et al., 2007). This mixed research approach is the third methodological paradigm after quantitative and qualitative research (Creswell, 1998). The embedded—mixed methods design typically involved one kind of data was embedded in the other. The form of the concrete presentation was a set of categorial data developed from the analysis of qualitative research and used as a framework in analyzing quantitative research data, so as to achieve data complementarity and enhancement (Jones et al., 2024; Nicolas et al., 2019; Stigsdotter et al., 2017). This study begins with semi-structured interviews with Arctic team members to gain insight into their perceptions and experiences, and to initially identify factors with restorative potential. Based on the factors initially identified, more Arctic team members were recruited to evaluate their satisfaction and fill in the Perceived Restorative Scale. The objective is to further identify factors with restorative potential. Finally, a combination of correlation analysis, principal component analysis and weight calculation are used to determine the design factors, dimensions and weight proportion that affect the restorative quality of the Arctic research station. The research framework is shown in Figure 1.

### 2.2 Research area

The Arctic region is the area within the Arctic Circle at 66° 34′ N near the North Pole, and it contains many islands distributed around the Arctic Ocean. Of these islands, the Svalbard archipelago in Norway (74°–81° N, 10°–35° E) is one of the most popular locations for Arctic research. In the summer, the average temperature in August is −3°C. Mosses and lichens grow in the archipelago, and wild geese and reindeer are common near the lakes. In winter, the average temperature in January ranges from −20 to −40°C. The annual polar day period is from April to August, and after the polar day, there are 10 cloudy and rainy days. The polar night period is from October to February.

**Figure 2:**
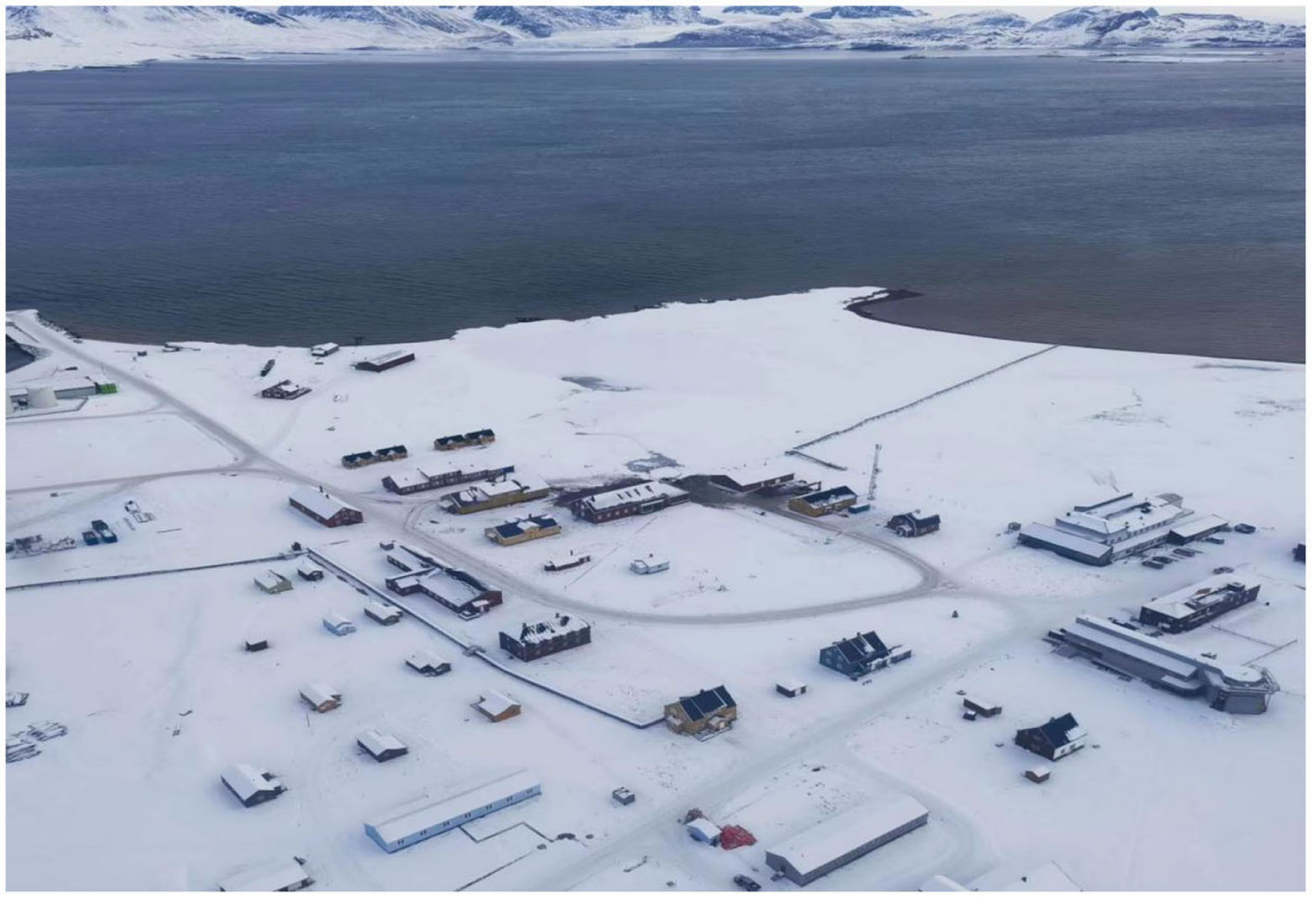
Ny-Ålesund. Provided by Arctic team members.

**Figure 3:**
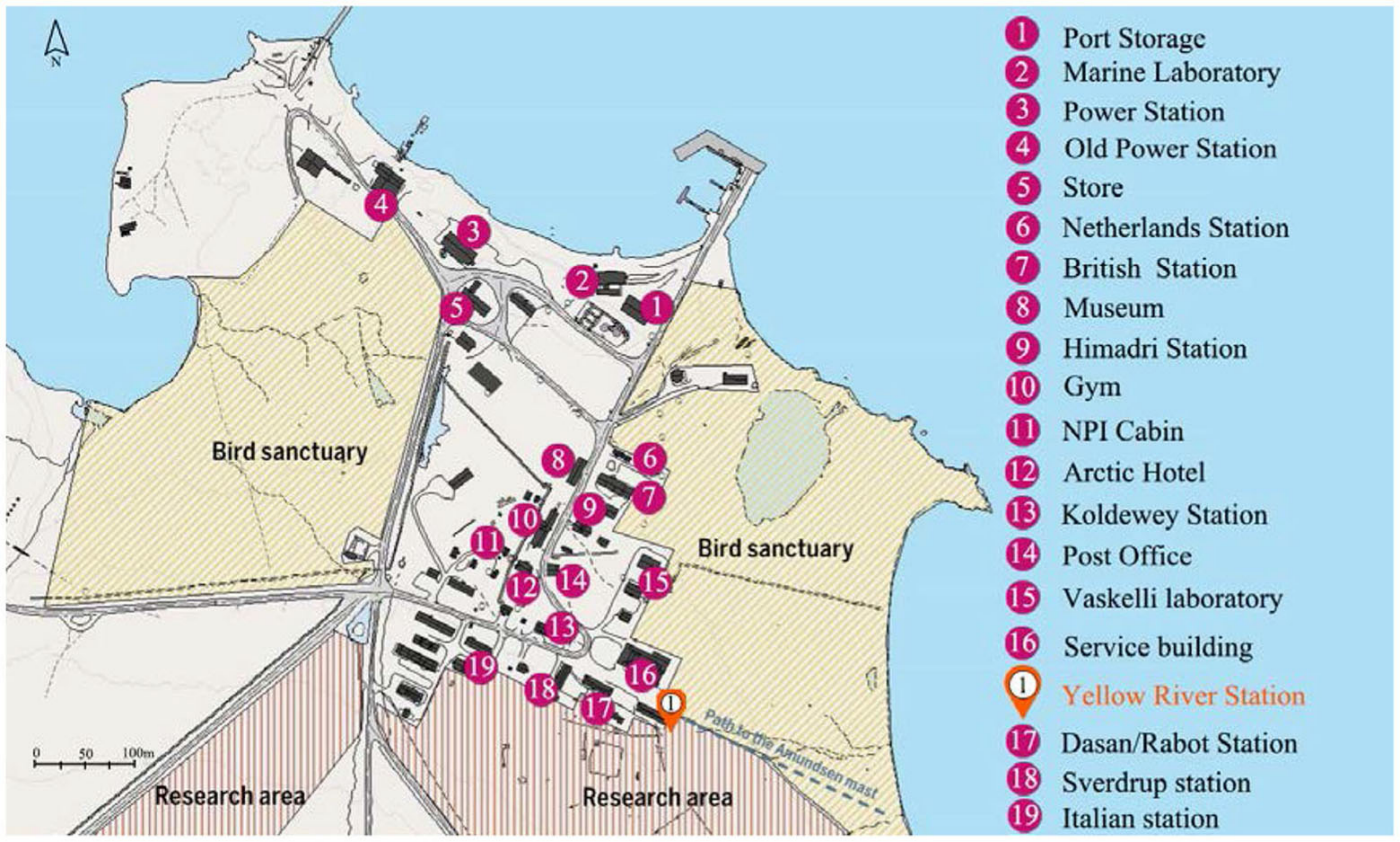
Ny-Ålesund facilities. Adapted from Choi et al. (2019).

**Figure 4:**
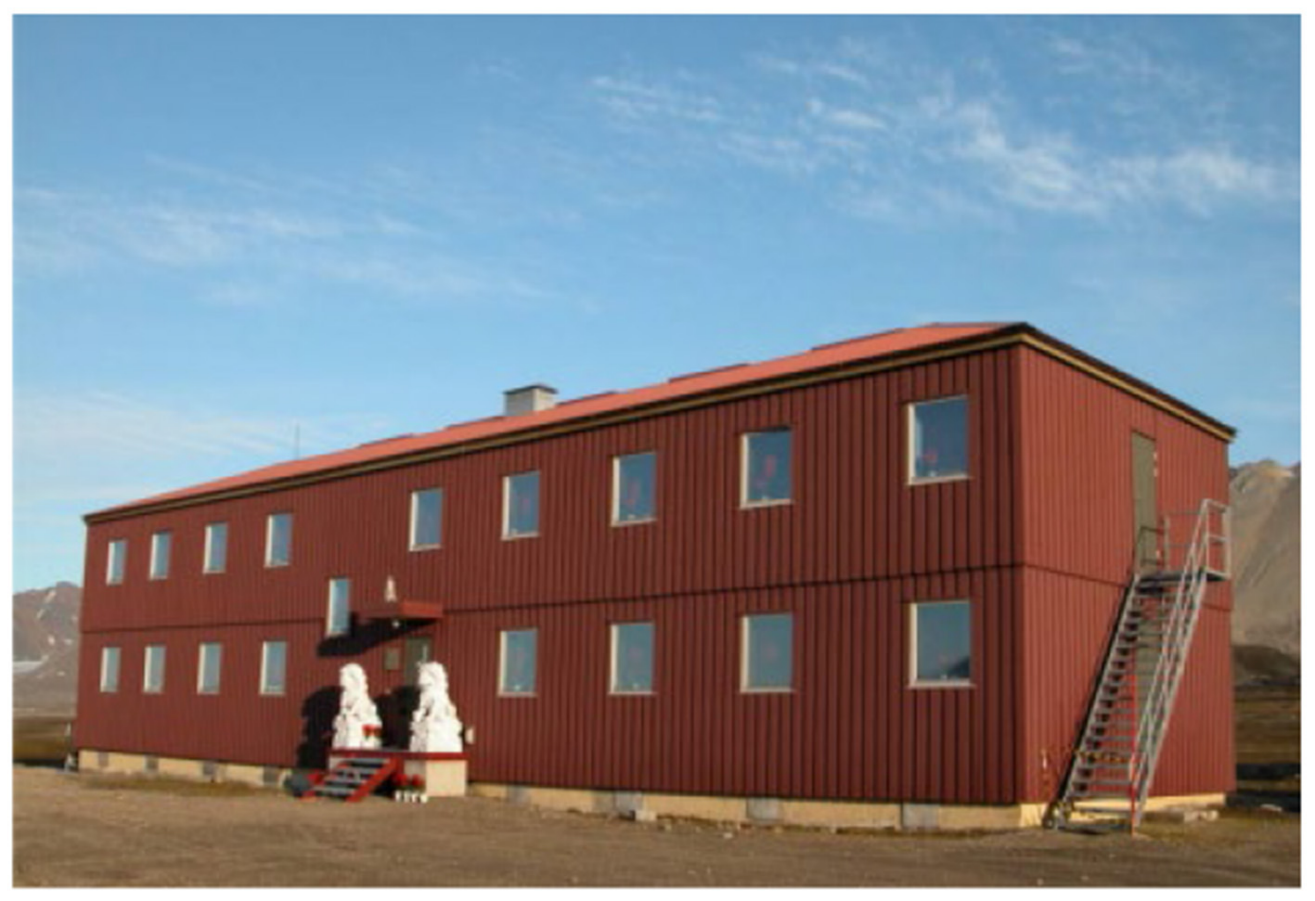
Arctic Yellow River Station. Reproduced from a https://blog.sina.com.cn/s/blog_79103bb90102whts.html.

**Figure 5:**
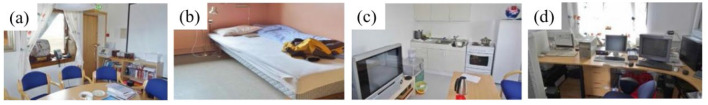
Indoor pictures of the Arctic Yellow River Station for **(a)** the meeting room, **(b)** a dormitory, **(c)** a small meeting room, and **(d)** the teams' office. Reproduced from a https://blog.sina.com.cn/s/blog_79103bb90102whts.html.

The site of this study was the Chinese Arctic Yellow River Station (78° 55′ N, 11° 56′ E). It is located in the town of New Olson (Figure 2), which is northwest of Svalbard, Norway. The distribution of the surrounding facilities is shown in Figure 3. The station is most populated in the summer; the scientific research station can accommodate 20–25 people working and living there at one time. The Arctic Yellow River Station is a small-scale building with a total area of approximately 500 m^2^ is shown in Figure 4 (Schiermeier, 2003). The station manager's room is a separate bedroom that includes a separate bathroom, and the rest of the team lives in separate or double bedrooms with a living area of approximately 10 m^2^. The public spaces include a multifunctional meeting room and a small dining room is shown in Figure 5. There is no medical treatment room inside the research station, and communication with the outside world is mainly done rough through sea and air transport.

### 2.3 Participants

In the first phase of research, team members of the Arctic expedition were selected to participate in the interview by a purposeful sampling method (Palinkas et al., 2015; Sandal et al., 2011; Sarris, 2017). By selecting participants who have worked in Arctic research stations and can provide a wealth of information, it is possible to ensure that participants have in-depth knowledge of specific phenomena and sufficient experience and insight into the research topic. The interviews were conducted from February 2022 to April 2022. The Arctic research team members were selected for the interview. Each participant gave informed written consent prior to the research activities. When the 10^th^, 11^th^, and 12^th^ subjects were interviewed, no new information about the restorative environmental perception of the Arctic research station appeared, so it can be judged that the data reached saturation (Popay et al., 1998). The interview information of these 12 subjects was taken as the interview sample, the demographics information of all interviewees is shown in Table 2. The sample consisted of 2 female participants and 10 male participants. The majority of respondents (58.34%) were aged between 26 and 35 years. Most researchers held a university degree or higher and were primarily engaged in research missions in the Arctic. The duration of their main expeditions typically ranged from 3 to 12 months.

**Table 2:** Population demographics of Arctic team members based on semi-structured interviews.

**Characteristic**		***n***	**%**
Gender	Male	10	83.33%
	Female	2	16.67%
Age	45+ years	1	8.33%
	36–45 years	4	33.33%
	26–35 years	7	58.34%
Educational background	Master or higher	6	50%
	Bachelor	6	50%
Cumulative time spent on scientific expeditions	3–6 months	5	41.67%
	6–12 months	5	41.67%
	12–24 months	2	16.66%

In the second stage of research, the quantitative survey also utilized a purposeful sampling method to recruit Arctic team members with working experience on the platform of Chinese Polar Research Network (Table 3). We utilized the G^*^Power software to calculate the statistical power and sample size, setting α to 0.05, d to 0.5, and the power to 0.95. The estimated required sample size was 52. Questionnaires were sent online via e-mail between April 2022 and September 2022. A total of 71 questionnaires were returned. We excluded the following two types of questionnaires with invalid responses: (1) more than two answers were missing, (2) the answers demonstrated a consistent tendency. Thus, data from 8 of the participates had to be excluded from the study due to the problems, the final sample included 63 participates. Each participant gave informed written consent prior to the research activities. The survey included 56 male participants and 7 female participants. Although the number of female researchers participating in expeditions has been gradually increasing, most studies indicate that male expedition members are more than their female counterparts (Temp et al., 2017). This phenomenon also reflects the gender composition of research teams, both past and in the present. The most common age group was 26–35 years, accounting for 44.4% (28 individuals) of the total sample, followed by 24 individuals aged 36–45, who represented 38.1% of the total. In addition to professional researchers, Arctic scientific research teams also include technical and logistical support personnel. Some team members had participated in multiple scientific expeditions to the Arctic. Therefore, the cumulative time each team member spent on scientific expeditions was calculated. The majority of members (22 individuals) had accumulated 12–24 months of expedition experience, representing 34.92% of the total sample.

**Table 3:** Population demographics of Arctic team members based on quantitative survey.

**Characteristic**		***n***	**%**
Gender	Male	56	88.89
	Female	7	11.11
Age	45+ years	11	17.50%
	36–45 years	24	38.10%
	26–35 years	28	44.40%
Educational background	Master or higher	43	68.30%
	Bachelor	15	23.80%
	Less than bachelor	5	7.90%
Cumulative time spent on scientific expeditions	0–3 months	7	11.11%
	3–6 months	10	15.87%
	6 months−12 months	4	6.35%
	12 months−24 months	22	34.92%
	24 months+	20	31.75%

### 2.4 Measures

#### 2.4.1 Qualitative research

The first stage was semi-structured interviews with interviewed lasted for an average duration of 30 min. Initially, an interview framework related to restorative benefits of indoor factors was developed based on existing literature studies (Zhang et al., 2024). This framework aimed to certify the effect of factors on the restorative of Arctic research stations environment, serving as the foundation and provided empirical support for a questionnaire survey on restorative environmental factors. The research extracted design factors based on literature review focusing on mental health and stress recovery. Table 1 presents the potential restorative benefits of these factors.

#### 2.4.2 Quantitative research

The satisfaction evaluation of environmental factors: The results of the qualitative experiment were used as a framework for evaluating the satisfaction of design factors in the quantitative research phase (Kitchen et al., 2024). According to the environmental factors extracted after the semi-structured interview coding, the 5-point Likert scale (1 = very dissatisfied to 5 = very satisfied) was used to collect participants' satisfaction with each environmental factor.

Perceived Restorative Scale (PRS): This study used the scale to explore their perceptions of the restorative quality of the research station environment (Hartig et al., 1998). The Restorative of Arctic research station was assessed within an adaptation of PRS scale composed by 16 items. This scale is divided into four dimensions, “being away” refer to physical and/or psychological, “being away” from demands on directed attention, divided into two items: (1) The station environment is an escape experience. (2) The station environment gives me a good break from my day-to-day routine. “Fascination” was an assumption of effortless attraction and attention, divided into six items: (1) The station environment has fascinating qualities. (2) My attention is drawn to many interesting things. (3) I would like to get to know this place better. (4) I want to explore the area. (5) There is much to explore and discover here. (6) I would like to spend more time looking at the surroundings. “Extent” contained richness in structure and content, divided into 3 items: (1) The station environment can make me extend a lot of good associations. (2) The station environment is in harmony with the surrounding environment. (3) The spatial functionality within the research station is chaotic. “Compatibility” meant that all my needs and interests can be met here, divided into 5 items: (1) I can do things I like in the station. (2) I can quickly adapt to life within the station. (3) I could find ways to enjoy myself in a place like this. (4) The way of life here, it fits my personality. (5) I feel safe in a station environment. Respondents indicate on a 7-point scale (0 = Not at all, 6 = Completely) the extent to which the given statement fits their experience of the Arctic research station environment.

### 2.5 Procedure

In the first phase, semi-structured interviews were used to guide the interviewees to express their opinions and views related to the research objectives. At the same time, in order to avoid interviewees being over-directed and not being able to express their ideas freely, the researcher participated as a listener and in an open and free flowing manner throughout the whole process. The time of semi-structured interview was generally limited to 20–40 min, and the interview needed to be recorded after obtaining the consent of the interviewees, and then translated into text for further analysis. In the second phase of quantitative study was conducted through a questionnaire, which consisted of three parts: a personal characteristics part (basic information such as age and gender), the satisfaction evaluation of environmental factors part, and Perceived Restorative Scale (PRS) part. Participants were recruited through China Arctic Online platform. Participants accessed the questionnaire by clicking on a link. After receiving the completed questionnaires, the questionnaires were screened and invalid questionnaires were eliminated.

### 2.6 Data collection and analysis processing

#### 2.6.1 Qualitative research

Twelve original interview transcripts were analyzed and coded using Nvivo11 analysis software (Kallio et al., 2016). The main purpose was to translate and summarize relatively casual and fragmented language of the participants into concise terms, and to refine the categories to clarify the concepts (Strauss and Corbin, 1998). When coding, we referred to the extraction results of environmental factors from relevant literature in Table 1, and read the interview text line by line, and bolded concepts related to environmental factors. This approach helped to condense and simplify the description of the environment. Table 4 presents a list of coded labels for two of the interview texts. After the initial definition, a total of 201 labels were obtained. After initial conceptualization and cluster classification, the initial primary concepts were formed (a). Subsequently, through literature comparison and concept elaboration, 59 secondary concepts (aa) were formed. Finally, the secondary concepts were logically grouped into 26 categories (A) related to environmental factors, including temperature, humidity, ventilation, noise, natural light, interior lighting, smell, color, room size, height, space layout, window size, furniture arrangement, specimens and decoration, personalization of the space, materials, separation of space, film viewing equipment, recreational facilities, sports equipment, space flexibility, space privacy, space interactivity, spatial connectivity and sparseness, partitioning functionality, and neatness of the environment, as shown in Table 4.

**Table 4:** Initial coding.

**Interview text (lable)**	**Initial concept (a)**	**Secondary concepts (aa)**	**Category (A)**
The height is only 2 meters, which is a bit depressing, and the windows are small…	a1. Height maybe 2 meters a2. Windows are small a3. A little depressing	aa1. Room height aa2. Window size aa3.Emotional depression	A1. **Room height** A2. **Window size** A3. Emotional depression
I am most afraid of this road from the Yellow River Station to the canteen; nothing can be seen at night, and polar bears appear. This section of the road is estimated to be 20 or 30 meters, but it is very dangerous…	a4. Scariest thing a5. Road from the station to the canteen a6. Nothing can be seen at night a7. Polar bear a8. 20 or 30 meters a9. Very dangerous	aa4. Fear aa5. Road from the station to the canteen aa6. Nothing can be seen at night aa7. Polar bear aa8. Very dangerous	A4. Fear of danger A5. Can't see environment in polar night A6. Polar bear A7. **Environmental safety**
Expedition members from all countries go to the restaurant to eat, and we usually talk there, in addition to talking about work—that is to say, where have we been, what happened…	a10. Expedition members from all countries a11. Eating at restaurant a12. Meeting and communicating	aa9. Team members aa10. Eating at restaurant aa11. Meeting and communicating	A8. Restaurant A9. **Interaction and communication**
In the summer, you can still see a little green, moss; there is also a vitality. After dinner, we will go out together to pick up some fossils of plants. We will also shoot the scenery here; it is very interesting…	a13. In the summer a14. A little green a15. Together to pick up some fossils a16. Shooting scenery a17. Very interesting	aa12. Picking up fossils aa13. Shooting scenery aa14. Interesting	A10. Picking up fossils A11. Shooting scenery A12. Interesting
There is a LAN in the conference room and office, but there is no dormitory; you can video or chat with your family when you are not busy, but it is a little inconvenient in such a public area…	a18. LAN in the conference and office but not in the dorm a19. Video or chat with your family a20. A little inconvenient in such a public area	aa15. Video or chat with your family online aa16. Inconvenient in public area	A13. Connecting with family online A14. Inconvenient in public area
If I want to exercise, I run in this building by myself. It's basically like a treadmill or something; you have to queue up…	a21. Exercise a22. Running in the building a23. Treadmill a24. Lining up	aa17. Exercise aa18. Running in the building aa19. Treadmill	A15. Sports space A16. **Sports equipment**
(A total of 201 original sentences)	……	…….	……

The bold values in this table represent concepts related to environmental factors that were identified during the coding process.

#### 2.6.2 Questionnaire survey

The data collected in the survey were analyzed in SPSS 26.0. The scale contains both forward and reverse coded questions to ensure the accuracy of the responses. For the reverse-coded items, we recoded them prior to data analysis (for example, converting “1” to “5,” “2” to “4,” and so on). Then, we assessed the scale's reliability. The reliability coefficient of satisfaction with environmental factors was 0.953 (>0.8), indicating strong reliability. The Kaiser-Meyer-Olkin (KMO) value was 0.819 (>0.7), χ^2^ = 1,655.579, and *P* < 0.001, which indicates the good validity of the factor. The scale of the restorative environment of the Arctic research station had a reliability coefficient of 0.951 (>0.8) with strong reliability. The KMO = 0.907, χ^2^ = 811.112, and *P* < 0.001, indicating the good validity of the scale.

In general, the restorative environment factors were difficult to assess directly. Team members tended to mix physical and psychological influences and assess features from perspectives other than that of restorative outcomes. Therefore, the evaluation of overall environmental recovery and the satisfaction factors were correlated to indirectly obtain the quality of recovery for each factor. Because the data were continuous variables, Spearman's correlation analysis was conducted (Gao and Zhang, 2021).

Additionally, to further integrate the factors of the Arctic research station, it is necessary to identify the internal structural characteristics of environmental factors that shares common properties through stratification and classification methods (Abdi and Williams, 2010). Therefore, the orthogonal dimension of design characteristics of restorative environmental factors of Arctic research station was extracted by principal component analysis.

Statistical weights, refer to the relative probability of a particular feature of a state, to determine the importance of the impact of each factor on the recovery of members. It involves calculating the proportion of each relevant index from the sum of all relevant indices.

## 3 Results

### 3.1 Restorative qualities of environmental factors

The design factors according to extracted after the semi-structured interview coding, team members' satisfaction with each design factor was collected using the 5-Likert subscale in descending order as follows in Table 5. A comparison of the team's results to the average design factor score is 3.606 indicated that which is at a relatively high level.

**Table 5:** Factors of satisfaction with the Arctic research station.

**Factor**	**Mean**	**SD**	**Factor**	**Mean**	**SD**
Temperature	4.411	0.443	Natural light	3.620	0.997
Personalization of space	4.030	0.668	Spatial connectivity and sparseness	3.612	0.992
Interior lighting	3.985	0.669	Materials	3.552	0.827
Room height	3.963	0.902	Separation of space	3.530	0.853
Color	3.844	0.897	Furniture arrangement	3.485	0.892
Sports equipment	3.799	0.892	Space privacy	3.455	1.029
Smell	3.791	1.084	Film viewing equipment	3.433	0.892
Window size	3.754	0.766	Recreational facilities	3.351	0.981
Space interactivity	3.747	0.935	Humidity	3.336	1.064
Ventilation	3.739	0.846	Space flexibility	3.329	0.911
Room size	3.732	0.947	Specimens and decoration	3.090	0.985
Functionality of partitioning	3.679	0.928	Noise	3.060	1.032
Space layout	3.657	0.947	Neatness of environment	2.769	1.223

### 3.2 Correlations between environmental factors and mental health rehabilitation

Spearman correlation analysis was performed on the perceived restorativeness of environmental factors. Table 6 shows that a Spearman correlation analysis examined the relationship between restorative perception and environmental satisfaction. It can be seen that, 24 design factors significantly correlate with restorative outcomes. Notably, privacy of the space exhibited the strongest correlation with Perceived Restorative Scale and its sub-dimensions (being away, fascination, extent, and compatibility), indicating it is one of the most critical factors affecting restorativeness. Other significant contributors included room height, color, and spatial interactivity. These findings imply that optimizing these indoor environmental characteristics can effectively enhance space comfort and human restoration. The extracted 24 design factors were labeled F1–F24 according to the order of the relevant values.

**Table 6:** Fact correlations between environmental factors and the restoration scale.

**Factor**	**Restorative score**	**Being away**	**Fascination**	**Extent**	**Compatibility**
Privacy of the space	0.685^**^	0.722^**^	0.662^**^	0.596^**^	0.616^**^
Room height	0.669^**^	0.648^**^	0.668^**^	0.485^**^	0.704^**^
Color	0.628^**^	0.633^**^	0.572^**^	0.451^**^	0.625^**^
Space interactivity	0.609^**^	0.539^**^	0.598^**^	0.490^**^	0.603^**^
Sports equipment	0.605^**^	0.537^**^	0.573^**^	0.440^**^	0.579^**^
Separation of space	0.595^**^	0.603^**^	0.634^**^	0.450^**^	0.514^**^
Functional zoning of the space	0.561^**^	0.465^**^	0.543^**^	0.506^**^	0.515^**^
Personalization of the space	0.537^**^	0.630^**^	0.570^**^	0.428^**^	0.449^**^
Space layout	0.520^**^	0.445^**^	0.536^**^	0.546^**^	0.448^**^
Space flexibility	0.506^**^	0.444^**^	0.502^**^	0.435^**^	0.475^**^
Materials	0.498^**^	0.540^**^	0.479^**^	0.364^**^	0.466^**^
Recreational facilities	0.472^**^	0.417^**^	0.449^**^	0.367^**^	0.426^**^
Furniture arrangement	0.457^**^	0.455^**^	0.502^**^	0.368^**^	0.371^**^
Room size	0.456^**^	0.563^**^	0.423^**^	0.330^**^	0.405^**^
Specimens and decoration	0.408^**^	0.369^**^	0.487^**^	0.358^**^	0.329^**^
Window size	0.406^**^	0.506^**^	0.388^**^	0.263^*^	0.353^**^
Film viewing equipment	0.390^**^	0.338^**^	0.369^**^	0.300^*^	0.355^**^
Neatness of the environment	0.382^**^	0.409^**^	0.401^**^	0.281^*^	0.351^**^
Temperature	0.382^**^	0.484^**^	0.394^**^	0.17	0.366^**^
Natural light	0.293^*^	0.384^**^	0.247	0.105	0.287^*^
Humidity	0.258^*^	0.264^*^	0.310^*^	0.207	0.149
Spatial connectivity and sparseness	0.248^*^	0.269^*^	0.253^*^	0.143	0.168
Interior lighting	0.234^*^	0.211	0.333^**^	0.166	0.223
Ventilation	0.222^*^	0.378^**^	0.243	0.074	0.174
Noise	0.217	0.326^**^	0.179	0.166	0.215
Smell	0.177	0.231	0.198	0.08	0.173

^**^Correlation is significant at the 0.01 level (two-tailed); ^*^correlation is significant at the 0.05 level (two-tailed).

### 3.3 Principal component analysis

Principal component analysis (PCA) is commonly used to classify the components of survey data, reduce dimensions, or generate new components. It is generally used to evaluate summative parameters, or for prediction and modeling purposes. In this study, PCA is applied to compress the data from the survey, resulting in component categories and the contribution degree related to the restorative perceptions. To determine suitability for PCA, a KMO test was initially conducted (KMO = 0.781, χ^2^ = 507.240, *P* < 0.001), and the results indicated the data was suitable for principal component analysis. As a result, factors with initial eigenvalues >1 were extracted, and five factors were identified. The explained variances of these five factors after rotation were 22.147%, 18.387%, 15.748%, 11.157%, and 7.018%. Their cumulative contribution rate of variance was 74.457%. The results show that the above statistics on environmental factors in the Arctic research station can explain most of the variability.

Table 7 shows the factor load using the maximum variance rotation. All study items corresponded to a common degree value higher than 0.4, indicating that the factors could extract information effectively. Principal component 1 consisted of color, room size, room height, space layout, materials, separation of space, furniture arrangement, specimen, and decoration; this component consisted of factors related to indoor conditions. Principal component 2 consisted of sports equipment, film viewing equipment, recreational facilities, space flexibility, and the functional zoning of the space; this component consisted of factors related to configuration of space. Principal component 3 consisted of temperature, humidity, ventilation, natural light, indoor lighting, and window size; this component consisted of factors related to the physical environment. Principal component 4 was composed of personalization of the space, space interactivity, and the privacy of the space, and was associated with the factor content clustering name for spatial perception. Principal component 5 was composed of spatial connectivity and the sparseness and neatness of environment; this component consisted of factors related to the factor content clustering name for the space safety. The extracted 19 design features were labeled F1 ~ F24 according to the order of the relevant values.

**Table 7:** Factor loading coefficients after rotation.

**Item**	**Factor loading coefficient**				
	**Factor 1 (22.147%)**	**Factor 2** **(18.387%)**	**Factor 3 (15.748%)**	**Factor 4** **(11.157%)**	**Factor 5 (7.018%)**
Color (F3)	0.546	0.311	0.526	0.287	−0.031
Room size (F14)	0.622	−0.221	0.383	0.433	0.17
Room height (F2)	0.604	0.137	0.252	0.534	0.079
Space layout (F9)	0.801	0.291	0.227	0.155	0.194
Materials (F11)	0.803	0.304	0.301	0.264	0.018
Separation of space (F6)	0.781	0.397	0.175	0.304	0.063
Furniture arrangement (F13)	0.765	0.41	0.209	0.195	0.22
Specimens and decoration (F15)	0.612	0.47	0.138	0.104	0.073
Sports equipment (F5)	0.232	0.722	0.288	0.399	0.063
Film viewing equipment (F17)	0.27	0.844	0.165	0.13	−0.017
Recreational facilities (F12)	0.227	0.850	0.264	0.214	0.152
Space flexibility (F10)	0.35	0.668	0.266	0.353	0.222
Functional zoning of the space (F7)	0.338	0.632	0.32	0.085	0.373
Temperature (F19)	0.176	0.167	0.670	0.3	0.185
Humidity (F2)	0.242	0.411	0.623	−0.194	−0.03
Ventilation (F24)	0.052	0.188	0.777	0.342	0.127
Natural light (F20)	0.169	0.356	0.479	0.235	0.439
Interior lighting (F23)	0.377	0.196	0.709	−0.108	0.028
Window size (F16)	0.472	0.178	0.606	0.32	0.038
Personalization of the space (F8)	0.373	0.21	0.326	0.474	0.319
Space interactivity (F4)	0.186	0.335	0.138	0.668	−0.072
Privacy of the space (F1)	0.376	0.296	0.05	0.653	0.26
Spatial connectivity and sparseness (F22)	0.049	0.036	0.022	0.102	0.841
Neatness of the environment (F18)	0.44	0.221	0.19	−0.108	0.457

Extraction method: principal component analysis. Rotation method: Varimax with Kaiser normalization. Rotation converged in 11 iterations.

To better summarize the factor classification, a second-stage principal component analysis was needed to further reduce the dimensionality within the first-level dimensions of indoor conditions, configuration of space, the physical environment, spatial perception and the space safety. The indoor condition factors were tested for reliability and validity. The results of the KMO test showed that KMO = 0.894, χ^2^ = 517.866, and *P* < 0.001, indicating that the data were well-structured and suitable for principal component analysis. Based on the existing literature and research practice, the initial eigenvalue was set to >0.6 to help ensure that the extracted factors have high explanatory power. Thus, two main components were screened (Table 8). Principal component 1 was composed of space layout, materials, separation of space, furniture arrangement, and specimens and decoration, this component consisted of factors related to appearance design, and was therefore named appearance design. The rotated sum of the squared principal component loadings was 45.754%. Principal component 2 was composed of color, room size, and room height, and consisted of factors related to the spatial scale; the sum of the squared principal component loadings after rotation was 36.303%.

**Table 8:** Factor loading coefficients after rotation.

**Item**	**Factor loading coefficient**	
	**Factor 1 (45.754%)**	**Factor 2 (36.303%)**
Space layout (F9)	0.777	0.462
Materials (F11)	0.728	0.597
Separation of space (F6)	0.799	0.51
Furniture arrangement (F13)	0.814	0.465
Specimens and decoration (F15)	0.886	0.132
Color (F3)	0.513	0.659
Room size (F14)	0.199	0.868
Room height (F2)	0.371	0.808

The five factors in configuration of space were tested for reliability and validity. The KMO results showed that the KMO = 0.827, χ^2^ = 333.468, and *P* < 0.001, which suggested their suitability for principal component analysis. The initial eigenvalue was set to >0.6, and two main components were screened (Table 9). Principal component 1 consisted of sports equipment, film viewing equipment, recreational facilities, and space flexibility, involving factors described interior facilities, and was thus named interior facilities, and the sum of the squared principal component loadings after rotation was 53.943%. Principal component 2 included the functional zoning of the space, which was named space function, and the sum of the squared principal component loadings after rotation was 35.042%.

**Table 9:** Factor loading coefficients after rotation.

**Item**	**Factor loading coefficient**	
	**Factor 1 (53.943%)**	**Factor 2 (35.042%)**
Sports equipment (F5)	0.714	0.53
Film viewing equipment (F17)	0.931	0.26
Recreational facilities (F12)	0.868	0.447
Space flexibility (F10)	0.682	0.593
Functional zoning of the space (F7)	0.319	0.923

The six factors of the physical environment were tested for their reliability and validity. The results showed that the KMO = 0.866, χ^2^ = 154.042, and *P* < 0.001, which indicated the factors' suitability for factor analysis. The initial eigenvalue was set to >0.6. Two main components were screened (Table 10). Principal component 1 was composed of temperature, ventilation, natural light, and window size, being associated with the factor related to outdoor environmental impact, and was therefore named outdoor environmental impact, and the rotated sum of the squared principal component loadings was 37.512%. Principal component 2 was composed of humidity and indoor lighting, being associated with the factor related to room adjustments, and was therefore named room adjustments, and the rotated sum of the squared principal component loadings was 32.658%.

**Table 10:** Factor loading coefficients after rotation.

**Item**	**Factor loading coefficient**	
	**Factor 1 (37.512%)**	**Factor 2 (32.658%)**
Temperature (F19)	0.581	0.51
Ventilation (F24)	0.656	0.517
Natural light (F20)	0.888	0.097
Window size (F16)	0.731	0.413
Humidity (F21)	0.143	0.873
Indoor lighting (F23)	0.375	0.701

The three factors of spatial perception were tested for their reliability and validity. A KMO test showed that KMO = 0.613, χ^2^ = 53.115, *P* < 0.001, which indicated their suitability for principal component analysis. The initial eigenvalue was set to be >0.6. Two main components were screened (Table 11). Principal component 1 was composed of personalization of the space and privacy of the space, and was described an individual's interactive perception of the environment, and was therefore named personal contact, the rotated sum of the squared principal component loadings was 52.522%. Principal component 2 was composed of space interactivity, which was named interaction, and the rotated sum of the squared principal component loadings was 36.763%.

**Table 11:** Factor loading coefficients after rotation.

**Item**	**Factor loading coefficient**	
	**Factor 1 (52.522%)**	**Factor 2 (36.763%)**
Personalization of the space (F8)	0.933	0.104
Space privacy (F1)	0.814	0.391
Interactivity of the space (F4)	0.206	0.969

There were only two factors in component 5, so no need to reduce its dimensionality. The dimensionality of the factors was further adjusted after two-dimensionality reductions. According to the similarity of the contents, the color (F3) in spatial scale (J2) was switched with the space layout (F9) in appearance design (J1); the space flexibility (F10) in interior facilities (J3) was moved under the dimension spatial function (J4) because it was more similar to the content of functional partitioning of the space; and the separation of space (F6) in appearance design (J1) was moved under the dimension of interaction (J8), which was more relevant to the creation of space. The classification of the adjusted factors is shown in Table 12.

**Table 12:** Classification of restorative characteristics.

**Primary component**	**Secondary components**	**Restorative factors**			
Indoor conditions (V1)	Appearance design (J1)	Color (F3)	Materials (F11)	Furniture arrangement (F13)	Specimens and decoration (F15)
	Spatial scale (J2)	Space layout (F9)	Room size (F14)	Room height (F2)	
Configuration of the space (V2)	Interior facilities (J3)	Sports equipment (F5)	Film equipment (F17)	Recreational facilities (F12)	
	Space function (J4)	Functional zoning of the space (F7)	Space flexibility (F10)		
Physical environment (V3)	Outdoor environmental influences (J5)	Temperature (F19)	Ventilation (F24)	Natural light (F20)	Window size (F16)
	Indoor adjustment (J6)	Humidity (F21)	Interior lighting (F23)		
Spatial perception (V4)	Personal contact (J7)	Personalization of the space (F8)	Privacy of the space (F1)		
	Interaction (J8)	Space interactivity (F4)	Separation of space (F6)		
Space safety (V5)	Space safety (J9)	Spatial connectivity and sparseness (F22)	Neatness of the environment (F18)		

### 3.4 Statistical weight calculation

Since this study will investigate the degree of contribution of each factor to environmental resilience, the correlation between the two is needed as the main basis for assigning weights. The statistical weights of the design features were calculated based on the recovery correlation index. The computed metric equation is as follows:


$$\begin{array}{l} W_{i}=\frac{c_{i}}{\sum_{i=24}c_{i}} \end{array}$$

*W*_*i*_ is the weight of factor i, *C*_*i*_ denotes the correlation coefficient between the environmental factor score and the environmental restorative score.

According to the principal component analysis results in Table 12, 24 factors were generalized as 5 principal components and 9 secondary components based on the results of PCA, after normalizing the weights, the calculation model of evaluation scores can be obtained as shown in Equation:


$$\begin{array}{l} V=\underset{n=24}{\sum }W_{n}F_{n}=W_{1}F_{1}+W_{2}F_{2}+W_{3}F_{3}+W_{4}F_{4}+\ldots \\ +W_{24}F_{24}=6.00\%F_{1}+4.70\%F_{2}+5.65\%F_{3}+4.97\%F_{4}+5.38\%F_{5}\\ +5.32\%F_{6}+4.57\%F_{7}+4.91\%F_{8}+4.16\%F_{9}+5.13\%F_{10}\\ +4.45\%F_{11}+4.32\%F_{12}+4.21\%F_{13}+6.01\%F_{14}+3.82\%F_{15}\\ +3.57\%F_{16}+3.56\%F_{17}+3.51\%F_{18}+3.46\%F_{19}+\\ 2.81\%F_{20}+2.53\%F_{21}+2.40\%F_{22}+2.28\%F_{23}+\;2.28F_{24} \end{array}$$

Based on the results of the second principal component analysis, it can be seen that the secondary-principal component factor J1 consists of F3, F11, F13, and F15; J2 consists of F9, F14, F2; J3 consists of F5, F17, F12; J4 consists of F7, F10; J5 consists of F19, F24, F20, F16; J6 consists of F21, F23; J7 consists of F8, F1; J8 consists of F4, F6; J9 consists of F22, F18 which can be derived:


$$\begin{array}{l} J_{1}=5.65\%F_{3}+4.45\%F_{11}+4.21\%F_{13}+3.82\%F_{15}\\ J_{2}=4.16\%F_{9}+6.01\%F_{14}+4.70\%F_{2}\\ J_{3}=5.38\%F_{5}+3.56\%F_{17}+4.32\%F_{12}\\ J_{4}=4.57\%F_{7}+5.13\%F_{10}\\ J_{5}=3.46\%F_{19}+2.28\%F_{24}+2.81\%F_{20}+3.57\%F_{16}\\ J_{6}=2.53\%F_{21}+2.28\%F_{23}\\ J_{7}=4.91\%F_{8}+6.00\%F_{1}\\ J_{8}=4.97\%F_{4}+5.32\%F_{6}\\ J_{9}=2.40\%F_{22}+3.51\%F_{18}\\ V=\underset{n=9}{\sum }W_{n}J_{n}=W_{1}J_{1}+W_{2}J_{2}+W_{3}J_{3}+W_{4}J_{4}+\ldots +W_{9}J_{9}=\\ 18.13\%J_{1}+14.87\%J_{2}+13.26\%J_{3}+9.70\%J_{4}+12.12\%J_{5}\\ +4.81\%J_{6}+10.91\%J_{7}+10.29\%J_{8}+5.91\%J_{9} \end{array}$$

Based on the results of the first principal component analysis, it can be seen that V1 consists of J1 and J2; V2 consists of J3 and J4; V3 consists of J5 and J6; V4 consists of J7 and J8, and V5 consists of J9. A recovery model based on the first principal component factor is established, as shown in Eq.


$$\begin{array}{l} V_{1}=18.13\%J_{1}+14.87\%J_{2}\\ V_{2}=13.26\%J_{3}+9.70\%J_{4}\\ V_{3}=12.12\%J_{5}+4.81\%J_{6}\\ V_{4}=10.91\%J_{7}+10.29\%J_{8}\\ V_{5}=5.91\%J_{9}\\ V=\underset{n=5}{\sum }W_{n}V_{n}=\;W_{1}V_{1}+W_{2}V_{2}+W_{3}V_{3}+W_{4}V_{4}+W_{5}V_{5}\\ =33\%V_{1}+22.96\%V_{2}+16.93\%V_{3}+21.2\%V_{4}+5.91\%V_{5} \end{array}$$

**Figure 6:**
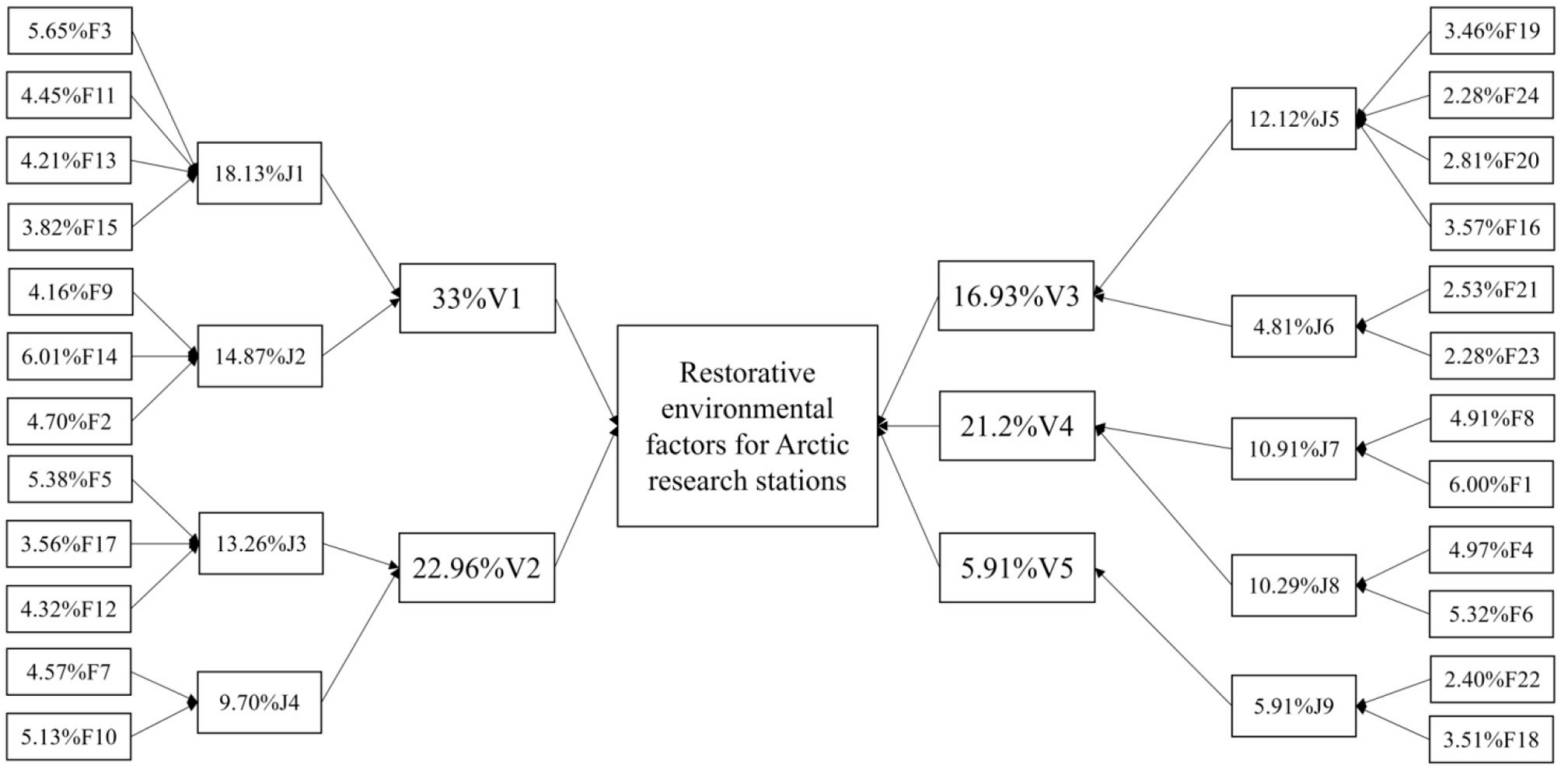
Model of the restorative quality of the Arctic Research Station environment.

The results show that five primary components were identified as indoor conditions, space configuration, physical environment, space perception, and space safety, and nine secondary components were identified as exterior design, space scale, interior facilities, space function, outdoor environmental impact, indoor regulation, personal contact, interaction, and space safety. According to the weight calculation, the two components of indoor conditions and space configuration have the highest weight. With the statistical weight calculations above, the model of the restorative quality of the Arctic Research Station environment was constructed in Figure 6.

## 4 Discussion

This qualitative study through semi-structured interview method screened 24 environmental factors in the Arctic research station that have a restorative effect on mental health. Finally, correlation analysis and principal component analysis were applied to determine the key environmental factors that had a restorative effect on the mental health of researchers at the Arctic research station. We found that these environmental factors are summarized into five first-level dimensions (indoor conditions, configuration of the space, physical environment, spatial perception, and space safety) and nine second-level dimensions (appearance design, spatial scale, interior facilities, space function, outdoor environmental influences, room adjustments, personal contact, interaction, and space safety). According to the weight calculation, indoor conditions and configuration of the space have the highest weight, indicating that they play a key role in improving the restorative perception of the Arctic research station.

In the extreme environment, the Arctic research station compared with the conventional environment, the primary medium that team members rely on for psychological restoration shifts from natural environment to the built environment's conditions in Arctic research stations. Under the circumstances, the basic function of the built environment becomes the primary guarantee of psychological recovery.

Based on the analysis of the dimensions and restoration perception of the Arctic research station, restorative design factors were compared at the factors level and the design should pay attention to high correlation factors. The indoor conditions dimension was further divided into two categories: appearance design and spatial scale. They were not only the basic building blocks of design, but also an important medium for stimulating and promoting environmental restoration. Appearance design is mainly related to the “fascination” dimension. In the Arctic research station environment, it is important to consider the specimens and decoration elements, as they incorporate features of local traditional culture (Davis, 2017). Team members, as mentioned in semi-structured interviews, express particular excitement about the specimens here. Therefore, this factor attracts interest with its unique and intriguing appearance, maintaining positive emotions and thus aiding in attention restoration (Suedfeld, 1991). Collapsible or deformable furniture as it enhances usability by dividing space in individual rooms more efficiently while also being attractive and practical for team members (Simon and Toups, 2014). The design element of material is related to “fascination.” Its restorative properties have been recognized as a way to alleviate stress in the workplace (Dreyer et al., 2018; Mangone et al., 2017). The main value of wood materials is that it can compensate for the lack of contact with nature through tactile and visual associations. Thereby, entering a more calm and relaxed state, which helps alleviate anxiety (Rice and Kozak, 2006; Tsunetsugu et al., 2007). The category of spatial scale is primarily associated with the “extent” dimension. The “extent” dimension pertains to whether the environment offers sufficient stimuli to capture team members' attention. The appropriate size, height and layout of the space can enable visual exploration by team members, and it can offer a sense of freedom and openness (Steel and Suedfeld, 2016).

The configuration of the space dimensions includes two categories: internal facilities and space functions. The internal facilities are mainly related to the two dimensions of “compatibility” and “fascination,” which mainly reflect the preferences of team members and the degree of support of space functions. Among them, the sports equipment, lounge, and recreational facilities factors significantly affected the mental health of the team members in our study, suggesting that people's preferences need to be matched to the space in their environment. During semi-structured interviews, team members emphasized that, outside of work, they primarily spend their time engaging in physical exercise and recreational activities. Therefore, some experienced team members would prepare to bring yoga mats and some common sports equipment to the Arctic research station. In polar environments, exercise has been identified as the most effective method for improving physical and psychological health. Facilities need to be provided in the environment to relieve mental fatigue and increase opportunities for physical activity, recreation, and leisure. The functionality of space is related to the dimensions of “fascination” and “compatibility,” indicating its ability to adapt to various activities and changes in needs. In the Arctic research station, flexibility is particularly crucial as team members may need to engage in multiple activities within limited space. Flexible spatial design reduces restrictions on space usage, offering more choices and possibilities, thereby enhancing team members' psychological adaptability and satisfaction (Shen et al., 2023).

In the physical environment dimensions, our research expands the current understanding of the restorative benefits that natural environments can provide. We have found that in polar environments, team members do not rely solely on plant landscapes for mental health restoration. Instead, they exhibit a preference for the extraordinary features of Arctic landscapes, such as glaciers and the aurora borealis (Hao et al., 2024; Joye and Bolderdijk, 2015). Furthermore, we have discovered that the synergistic interaction between natural elements (such as temperature and natural light) and artificial adjustments has a significant potential to positively influence the mental health of team members. This synergy has developed into a unique form of natural compensation adapted to the polar environment. Specifically, the physical environment dimension included two categories, outdoor environmental influences and indoor adjustment. The elements of outdoor environment are mainly related to the dimension of “being away.” Positive interaction with natural elements has long been recognized as a key method for distancing oneself from stressful environments and enhancing restoration. At the same time, the Arctic's scenery such as the aurora borealis, glaciers, and expansive snowfields, provide a unique natural experience for the team members. These landscapes not only create a psychological sense of “being away” from everyday surroundings but also stimulate the team members' interest. Therefore, the size of the window can be appropriately increased (Tregenza and Wilson, 2011). When the view is open and the polar landscape through the window can autonomously capture team members' attention, thus promoting psychological recovery. Meanwhile, artificial natural compensation shows unique advantages. As the conclusion of Caron-Rousseau study, accurate control of indoor temperature, air quality and indoor lighting are important factors in the mental health of the team members (Caron-Rousseau et al., 2022). It not only improves the comfort of the environment, but also enhances the effect of psychological recovery (Friborg et al., 2014).

As for the dimension of spatial perception, we found a conclusion consistent with Yan's research through the combination of qualitative and quantitative methods: in the polar environment, space privacy and control are more important for the restorative potential of Arctic research stations (Yan and England, 2001). This difference is due to the uniqueness of the polar environment, where people need to feel more in control to alleviate psychological stress. Specifically, the spatial perception dimension included two categories, personal contact and interaction. Personal contact is mainly related to the dimension of “being away,” especially a psychological sense of “being away.” Creating an environment in which team members can interact and communicate is critical to fostering unity and harmonious relationships. Furthermore, particularly the balance between private and shared spaces, plays a pivotal role in enhancing occupants' sense of control and privacy, which are essential for mental recovery (Binsted et al., 2010; Jaksic et al., 2019). This study not only confirms previous research findings, but also methodologically bridges gaps in earlier qualitative studies. It also echoes Hartig's discussion on the variability of resources in the built environment (Hartig, 2021). In the second category, the interactivity of space is mainly related to the dimension of “fascination,” Design that fosters social interaction and provides multi-functional areas can enhance the psychological wellbeing of the team members. Therefore, when designing Arctic research stations, such as by establishing public activity spaces that encourage social activities. The limited space of the research station can be enhanced by the clever zoning design, which would create a sense of more space and improve people's interactions, and enhance the overall working atmosphere. It contributes to the satisfaction and positive mood of team members and also provides a good working environment for the successful execution of scientific missions.

The space safety in the Arctic environment also has restorative potential, a factor that has not been included in previous studies. This is a consideration of safety standards for scientific buildings. Safety is considered a fundamental prerequisite for the restorative effects of Arctic research station environments, being crucial for creating an environment conducive to restoration. A clean, well-maintained, and safe environment with a layout that promotes connectivity and a sense of spaciousness can reduce stress and anxiety of team members caused by the environment, fostering a sense of wellbeing and relaxation, which are key components of restorative experiences.

The limitations of this study are as follows: (1) The retrospective self-report survey of the team members could be biased due to the limits of their memory. (2) The participants were all Chinese expedition members, and the findings may not be generalizable to people from different countries.

## 5 Conclusions

This study systematically identified and analyzed the environmental factors that promote the psychological recovery restorative quality of Arctic research team members. Through an embedded mixed-method approach that combined semi-structured interviews and questionnaires, we constructed a weight model for the restorative quality of the Arctic research station environment. This study extends the restorative environment theory to the context of extreme environments. From the perspectives of stress alleviation and psychological recovery, we proposed insights into various factors. The findings provide a theoretical foundation and valuable insights for the architectural design of Arctic research stations. These findings are not only applicable to Arctic research stations but also have implications for architectural design in other extreme environments (such as deep-sea workstations and space stations). Furthermore, there is a need for further exploration and comparison of the differences in restorative needs among different international teams to develop more targeted and adaptive design strategies. Through these findings, we hope to provide a more comprehensive perspective for the design of research stations, thereby promoting the psychological health of research team members.

## Data Availability

The original contributions presented in the study are included in the article/supplementary material, further inquiries can be directed to the corresponding author.
